# The Use of Artificial Intelligence in ECG Interpretation in the Outpatient Setting: A Scoping Review

**DOI:** 10.7759/cureus.94113

**Published:** 2025-10-08

**Authors:** Raghavee Neupane, Oksana Denis, Mollie Goudy, Mark Lezanic, Brandon Nguyen, Matthew Nguyen, Jared Plasencia, Victor Hugo Spitz, Vanessa Stalls, Abraham Yacaman, Robin J Jacobs

**Affiliations:** 1 Medicine, Dr. Kiran C. Patel College of Osteopathic Medicine at Nova Southeastern University, Fort Lauderdale, USA; 2 Medical Education, Dr. Kiran C. Patel College of Osteopathic Medicine at Nova Southeastern University, Fort Lauderdale, USA

**Keywords:** arrhythmia detection, artificial intelligence, cardiac amyloidosis, cardiovascular disease, convolutional neural network, deep learning, diagnostic accuracy, electrocardiogram, machine learning, outpatient care

## Abstract

Cardiovascular disease remains the leading cause of death across all demographics globally. The 12-lead ECG is a key diagnostic tool for early detection; however, its interpretation is complex and prone to error, particularly in outpatient settings. AI, especially deep learning models such as convolutional neural networks (CNNs), has shown potential in improving ECG interpretation, but its effectiveness outside hospital environments remains underexplored. This scoping review summarizes the use of deep learning algorithms for ECG interpretation in outpatient settings, focusing on diagnostic accuracy and clinical utility. A systematic, comprehensive literature search was conducted using EMBASE, Ovid MEDLINE, and Web of Science. Included studies examined AI-based ECG interpretation in outpatient care. Studies set in emergency departments, case reports, reviews, and purely theoretical AI models were excluded. Data on study design, AI methods used, and clinical outcomes were extracted. Study quality was assessed using the Joanna Briggs Institute Critical Appraisal tools. Findings from this review demonstrate that AI-assisted ECG interpretation, particularly using deep learning, improves diagnostic accuracy and enables earlier detection of cardiac conditions. These tools show the greatest potential in resource-limited outpatient settings, helping reduce misdiagnosis rates and easing the burden on specialists. However, limitations persist, including small and homogenous sample sizes, and a tendency for some algorithms to overdiagnose. Issues such as data standardization, lack of diversity in training datasets, and limited external validation by cardiologists hinder real-world application. Larger, more representative datasets and robust clinical testing are essential to enhance the generalizability and reliability of these tools. AI integration into outpatient ECG interpretation holds significant promise for improving cardiovascular diagnostics, particularly in underserved areas. Future research should prioritize real-world validation across diverse populations and emphasize cardiologist oversight to ensure clinical safety and effectiveness.

## Introduction and background

Cardiovascular disease

Cardiovascular disease (CVD) remains the leading cause of mortality worldwide, affecting both women and men across various racial and ethnic backgrounds [1,2]. A myocardial infarction (MI) occurs every 40 seconds in the United States [3], underscoring the need for improved strategies beyond traditional risk factor management, such as quitting smoking, controlling blood pressure, and managing high cholesterol [2]. Despite preventive efforts, diagnostic gaps persist in the interpretation of ECGs, where many healthcare professionals report inadequate training and low confidence [4].

12-lead ECG

The 12-lead ECG is a widely used and accessible tool for diagnosing cardiac abnormalities such as arrhythmias and acute coronary syndromes [5]. When interpreted correctly and promptly, it can prevent adverse outcomes [6]. ECG interpretation has been shown to decrease negative consequences (e.g., incorrect diagnoses, delayed treatment) and mitigate risks (e.g., misinterpretation of cardiac events, failure to recognize MI) in patients with cardiac symptoms, while identifying the need for emergency interventions [6]. Although ECGs are widely available and relatively inexpensive compared to other diagnostic modalities, studies report low proficiency among trainees, as well as inconsistencies in algorithm-based diagnoses due to non-standardized criteria [4,5]. These limitations are more pronounced in outpatient and rural settings, where access to specialists is limited, thereby increasing reliance on potentially flawed automated interpretations [7]. Inaccurate ECG readings can result in delayed treatment, unnecessary interventions, or fatal misdiagnoses.

Although a 12-lead ECG may identify the source of palpitations in about 40% of cases [8], intermittent conditions often go undetected without extended monitoring. Ambulatory ECG tools, such as Holter monitors, are therefore helpful in outpatient settings for detecting arrhythmias, particularly intermittent or transient ones, that are often missed by standard resting ECGs [9,10]. As hospitals seek to reduce inpatient stays, outpatient diagnostic tools must evolve to detect insidious cardiovascular conditions early [11].

AI

AI is an umbrella term encompassing technologies capable of mimicking human cognitive functions such as learning, reasoning, and problem-solving [12,13]. AI in healthcare utilizes techniques such as Machine Learning (ML), Deep Learning (DL), and Natural Language Processing (NLP) to analyze complex datasets and improve diagnostic precision [13,14].

ML algorithms are further subdivided into supervised, unsupervised, and reinforcement-based learning, each playing a unique role in healthcare, from risk prediction to imaging and treatment planning [12,13]. Supervised learning uses labeled data to make predictions, such as diagnosing cancer through imaging and predicting outcomes from EHR [12,13]. Unsupervised learning identifies hidden patterns without predefined labels, helping personalize treatments or detect disease risks from genetic data [12,15]. Reinforcement learning uses feedback to optimize decisions in real time, such as guiding robotic-assisted surgery.

DL models, an advanced form of ML, excel at analyzing large and complex datasets, making them particularly effective in image interpretation, genomics, and EHR analysis [13]. NLP converts unstructured clinical text, such as provider notes, into structured, machine-readable data and often complements ML models to improve diagnostic and treatment recommendations [12,14]. These AI subsets have demonstrated accuracy in anomaly detection, supporting earlier and more precise diagnoses [13]. AI has been shown to enhance clinical decision-making, reduce diagnostic errors, and deliver real-time alerts that help predict patient outcomes [12,14].

AI in ECG interpretation for outpatient settings

Recent advancements in AI present new opportunities to address the longstanding limitations of ECG interpretation, particularly in outpatient environments where diagnostic resources are limited [16]. AI integration has been shown to improve the detection of arrhythmias, structural heart disease, and other abnormalities that may elude traditional interpretations [17]. DL models have achieved diagnostic accuracy comparable to expert cardiologists when analyzing ECGs, even with single-lead data [18,19]. Likewise, AI has demonstrated capabilities such as estimating patient age and sex, as well as predicting future cardiac events such as atrial fibrillation and ventricular dysfunction [20,21].

By increasing diagnostic precision and efficiency, AI, especially ML and DL, holds the potential to improve cardiovascular outcomes in outpatient settings. However, despite growing evidence of its utility, relatively few studies have explored its use in outpatient ECG interpretation, highlighting a gap in current research.

Objective

This review aimed to summarize the most recently published literature on how AI technologies can assist with ECG interpretation in outpatient settings and to assess their impact on diagnostic accuracy and clinical outcomes.

Review Question

The review question is based on the Population, Concept, and Context (PCC) framework, establishing P for patients undergoing ECG testing, C for the capabilities of AI technologies to interpret ECGs, and C for outpatient settings. The main review question was: “How does the use of AI in outpatient settings help interpret ECGs and affect clinical outcomes?”

## Review

Methods 

A comprehensive literature search was performed using EMBASE, Ovid MEDLINE, and Web of Science. Key search terms included “artificial intelligence,” “ECG interpretation,” and “outpatient setting,” combined using Boolean operators (“AND,” “OR”). Only English-language articles published between January 1, 2014, and August 1, 2024, were included. The search was limited to peer-reviewed, published studies. The Nova Southeastern University (NSU) library database was utilized to access databases and full-text articles.

Eligibility Criteria

Studies were eligible for inclusion if they met the following criteria: the study design consisted of cohort studies, case-control studies, randomized controlled trials, validation studies, diagnostic accuracy studies, quality improvement projects, or case series. The study population included patients of any age who underwent ECG testing in an outpatient setting. The intervention of interest was the use of AI for ECG interpretation, and eligible studies reported outcomes related to diagnostic accuracy and/or clinical outcomes.

Studies were excluded if they focused on inpatient or emergency settings, involved ECG testing for non-cardiac purposes (such as preoperative assessments), or were theoretical studies, case reports, reviews, or editorials.

Study Selection and Critical Appraisal of the Evidence

**Figure 1:**
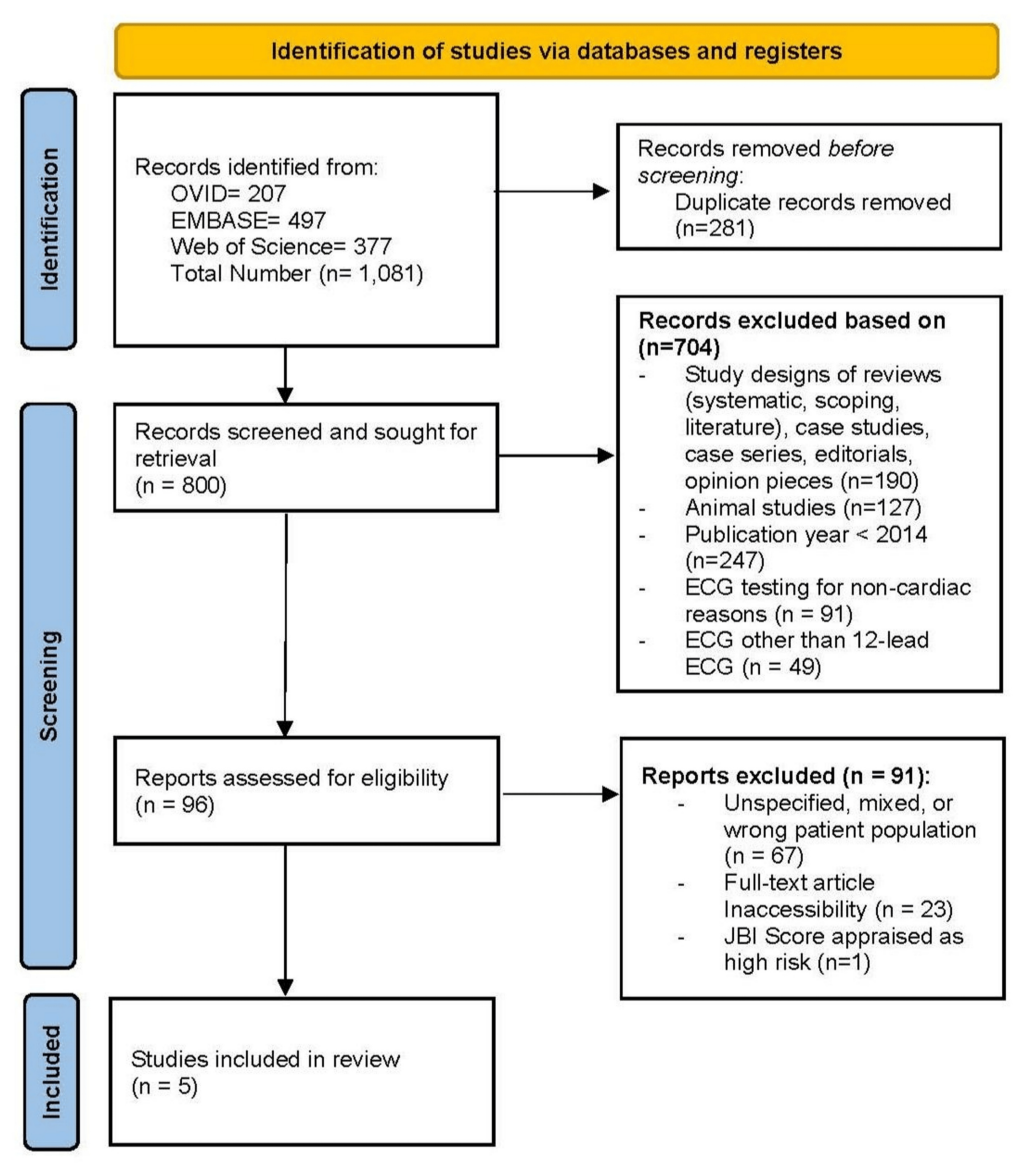
PRISMA flow diagram of the search and selection process. PRISMA: Preferred Reporting Items for Systematic Reviews and Meta-Analyses.

The search yielded 1,081 studies; after removing 281 duplicates, 800 unique articles remained. Title and abstract screening (Tier 1) by 10 independent reviewers reduced this to 96 full-text articles (Tier 2), of which six met the inclusion criteria. Quality assessment using the Joanna Briggs Institute Critical Appraisal Tools categorized the risk of bias as low (>70%), moderate (50-70%), or high (<50%) [22]. Two blinded reviewers assessed each study, resolving disagreements through discussion or consultation with a third reviewer. One high-risk study was excluded, leaving five studies in the final analysis. The selection process is illustrated in the PRISMA-ScR flow diagram (Figure 1).

Results 

Overview of Included Studies

Most of the reviewed studies used retrospective cohort, observational, or multicenter designs, focusing on AI-driven ECG interpretation to detect conditions such as cardiac amyloidosis (CA) and arrhythmias [23-27]. Sample sizes varied widely, ranging from a small, homogenous cohort of 63 African American adults [24] to over 336,000 ECGs from 142,020 participants [25].

More than half of the studies developed and trained deep learning models such as convolutional neural networks (CNNs) or echo state networks (ESNs), while others employed AI algorithms for ECG interpretation [24,26]. Regardless of the model type, all studies demonstrated improvements in diagnostic accuracy and reductions in human error. However, authors emphasized the need for further validation, particularly across different cardiac conditions and populations. The number of studies examining the use of AI in healthcare settings has grown in recent years, although many still rely on retrospective data [28].

Comparing AI Performance With Standard Diagnostic Techniques

Several studies showed that AI models, particularly those using deep learning, can match or even outperform traditional diagnostic methods and expert clinicians. For instance, a deep learning model for ECG triage achieved high accuracy (C-statistic = 0.93) compared to traditional computer-based ECG analysis tools [25]. In another study, a combination of a 2D-CNN ECG and a 3D-CNN echocardiogram model was used, and this dual approach led to improved diagnostic accuracy and a higher positive predictive value (PPV) [23].

Neural Networks and Algorithms for ECG Analysis

Neural networks and advanced algorithms have demonstrated strong utility in analyzing ECG data. A CNN model developed in 2020 achieved 99.15% accuracy in detecting normal sinus rhythm and atrial fibrillation [27]. Another CNN model detected myocardial infarction (MI) with 93% accuracy and successfully triaged patients based on ECG abnormalities in the ST segment, T wave, and lead II [25]. A summary of the studies included in this review is presented in Table 1.

**Table 1:** Summary of the studies included in the review on AI-enhanced ECG interpretation for outpatient cardiac diagnostics. AI: Artificial Intelligence; AI-ECG: Artificial Intelligence Electrocardiogram; CA: Cardiac Arrest; CNN: Convolutional Neural Network; 2D-CNN: Two-Dimensional Convolutional Neural Network; DNN: Deep Neural Network; ECG: Electrocardiogram; ECHO: Echocardiogram; hsECG: High-Sensitivity Electrocardiogram; LVDD: Left Ventricular Diastolic Dysfunction; LVEF: Left Ventricular Ejection Fraction.

Author	Aim of Study	Study Design	Study Population	Methods	Limitations	Key Findings
Goto S et al. (2021) [23]	Develop and validate a machine learning-based AI model for detecting CA using ECG	Retrospective multicenter	9,209 adults (CA = 597; controls = 8,612)	Developed 2D-CNN ECG models using data from five institutions; compared model performance against expert cardiologists	Potential mislabeling of controls due to underdiagnosis; limited generalizability without broader clinical data	Models showed strong predictive ability in detecting CA (C-statistic: 0.85-0.91)
Harmon DM et al. (2022) [24]	Assess AI-ECG for cardiovascular screening in community-based settings	Observational	63 African American participants	12-lead ECGs analyzed by AI to predict age, sex, and LVEF; compared with ECHO	Small sample size with LVEF <50% (n = 2); limited correlation with clinical outcomes	AI-ECG may enhance community-based cardiovascular screening programs
van de Leur RR et al. (2020) [25]	Validate a deep convolutional neural network (DNN) for automated triage with 12-lead ECGs	Observational	AI trained on 336,835 ECGs from 142,040 patients and validated on 984 ECGs	Deep CNN trained on ECGs to detect acute cardiac abnormalities; compared to cardiologists	Validation involved only 2-3 cardiologists and was limited to acute conditions	DNN ECG interpretation achieved high diagnostic accuracy
Whitman M et al. (2024) [26]	Test AI for detecting early LVDD from 12-lead ECGs with wavelet signal processing (hsECG)	Prospective observational	464 patients with chest pain	Compared 12-lead ECG and hsECG results with ECHO to identify early LVDD	Low-risk population; single site; possible selection bias	Machine learning algorithms interpreting hsECG showed high sensitivity and low specificity; potential for overdiagnosis and early detection of LVDD
Zhang X et al. (2020) [27]	Use CNNs to detect life-threatening arrhythmias from ECGs	Retrospective cohort	Data divided into training (n = 259,789) and testing (n = 18,018) sets	CNN trained on 259,789 ECGs and validated on 18,018 ECG signals targeting arrhythmias and normal rhythms	Data imbalance, unequal signal lengths, and underrepresented labels	CNN model achieved an overall accuracy of 95%, with 99.15% accuracy for detecting normal rhythm and atrial fibrillation

Early Detection and Screening

AI has been shown to enhance the early diagnosis of cardiac conditions such as left ventricular diastolic dysfunction (LVDD), Brugada syndrome, and arrhythmias. A CNN was developed and trained using ECG data, echocardiogram data, and a combination of both for the early detection of CA [23]. The results were promising, with predictive scores (C-statistics) between 0.85 and 0.91. The 2D-CNN trained only on ECG data demonstrated high sensitivity (between 52.4% and 71%) and enabled the detection of CA nearly a full year before formal clinical diagnosis by cardiologists. The 3D-CNN trained on echocardiograms achieved 96% accuracy, outperforming expert cardiologists. When the 2D-CNN was used as the initial screening tool and the 3D-CNN was used for confirmation, diagnostic accuracy and efficiency improved significantly [23]. AI-ECG technology was also tested in community settings, such as churches, and showed potential for screening reduced heart function, particularly in underserved areas, after addressing certain limitations [24].

Discussion 

This systematic review examined the applications of AI algorithms in interpreting ECGs for outpatient cardiac diagnostics. The findings support the growing consensus that AI algorithms enhance ECG interpretation and improve clinical outcomes. While previous research has largely focused on inpatient and acute care settings, this review highlights the potential of AI as a valuable tool in outpatient and resource-limited environments.

Improved Accuracy With AI Algorithms

Across all studies, AI models, particularly deep learning algorithms such as CNNs and ESNs, consistently outperformed traditional ECG interpretation methods and, in some cases, even expert clinicians [23,25]. These improvements are especially meaningful given the global burden of cardiovascular disease [1,2]. Enhanced diagnostic accuracy enables earlier and more reliable identification of cardiac conditions, potentially reducing morbidity and mortality [29].

Many clinicians, especially in underserved areas, report limited confidence in ECG interpretation due to gaps in training [4,6]. This skill gap contributes to diagnostic disparities between urban and rural populations, where access to cardiologists remains limited [7,30]. Integrating AI into outpatient care could bridge this gap by providing decision support to non-specialists and improving outcomes [5]. For example, Harmon DM et al. (2022) demonstrated the utility of AI-ECG tools in community-based screenings, particularly in underserved populations [24].

Early Detection Using AI Algorithms

Several studies highlighted AI’s ability to detect cardiac conditions, such as arrhythmias and CA, well before conventional diagnosis is possible [23,25,27]. One CNN model predicted disease onset nearly a year in advance, outperforming expert cardiologists [23]. Others demonstrated AI algorithms’ utility in triage, accurately identifying and prioritizing acute conditions [25]. Given that MI occurs every 40 seconds in the United States, timely recognition of risk factors such as arrhythmias is critical [3]. AI’s capacity to flag high-risk patterns early could reduce delays in care and improve survival rates [29,31].

Limitations

While the findings reviewed offer promising insights into the application of AI in cardiovascular diagnostics, several limitations emerged across the studies. Common challenges included small sample sizes [24], potential mislabeling of control groups [23], and the lack of standardized external validation, which limits confidence in model performance across diverse, real-world clinical settings [25]. For instance, one study used an AI algorithm to screen for reduced heart function in a community setting, but the small and homogeneous sample of 63 African American adults limited its generalizability. Among the participants, only two had reduced heart function, and the AI model correctly identified just one, yielding a sensitivity of 50%. However, with such a limited subset, no meaningful conclusions could be drawn regarding clinical efficacy [24]. These findings underscore the need for testing AI models across more diverse and representative populations before implementation in outpatient settings.

Another recurring issue was the tendency of AI models, particularly deep learning systems, to overdiagnose. For example, in detecting early LV diastolic dysfunction using wavelet signal-processed ECGs, one algorithm achieved a sensitivity of 84.5% but a lower specificity of 47.9% [26]. Similarly, a 2D-CNN model trained exclusively on ECGs reached up to 71% sensitivity but had a PPV of only 3.9%. When this model was combined with a 3D-CNN trained on echocardiograms, the PPV improved to 32.7%, suggesting that multimodal approaches may help mitigate overdiagnosis and enhance reliability [23].

Generalizability also remains a concern. Many AI models have yet to prove effective across broader populations or different cardiac pathologies. Although large datasets offer the promise of more robust model training, they are not without drawbacks. Several studies encountered standardization issues, such as ECGs with unequal signal lengths or class imbalance, that required additional data preprocessing, including frame division and modification [27]. Furthermore, rare or underdiagnosed conditions like CA are often underrepresented, increasing the risk of mislabeled controls and reducing diagnostic accuracy [23].

These limitations highlight the importance of continued refinement, validation, and inclusivity in dataset design to ensure that AI tools can be safely and effectively integrated into real-world cardiovascular care.

Implications

The studies collectively suggest that AI-based ECG analysis holds significant potential for broader applications beyond the specific conditions tested in the reviewed studies. The possibility of generalizing AI to other cardiac diseases and integrating it into routine clinical practice was a recurring theme, although further research is needed to optimize its application. Future research could explore expanding the use of AI algorithms as a screening tool in underserved community settings to assess not only reduced heart function but also other acute cardiac conditions. The combined use of CNN-based ECG analysis for detecting abnormalities during triage, along with AI screening tools, could help bridge the gap between urban and rural mortality rates from cardiac conditions. Expanding study populations from single communities to a broader range of underserved and rural groups may also improve the confidence and reliability of AI algorithms in screening for reduced heart function, while testing their performance in populations experiencing diverse environmental and physiological challenges. Moreover, the implementation of AI interpretation in wearable heart-monitoring devices, such as Holter monitors, is of particular interest given the increasing prevalence of portable ECG use in outpatient populations.

## Conclusions

This review highlights the potential of AI in advancing cardiovascular care, particularly in ECG interpretation. AI technologies have the capacity to enhance diagnostic accuracy, enable earlier detection of subtle abnormalities, and alleviate the workload of healthcare providers. Several studies have demonstrated that AI models can match, and, in some cases, exceed, expert-level performance by identifying patterns that may be overlooked during human interpretation. As cardiovascular disease remains the leading cause of mortality worldwide, the integration of AI offers a promising path forward, especially for rural and underserved communities where access to high-quality cardiac care is limited. By bridging these disparities, AI has the potential to act as an equalizer in cardiovascular health outcomes. However, to fully realize this potential, key challenges must be addressed, including limitations in available datasets, the need for rigorous clinical validation by cardiologists, and concerns about overdiagnosis. Addressing these challenges will be essential to ensuring safe, effective, and equitable implementation. With continued research and thoughtful integration into clinical workflows, AI can become a transformative tool in the early identification and management of cardiac conditions, ultimately improving care delivery and outcomes across diverse healthcare settings.
